# Primary stenting for femoropopliteal peripheral arterial disease: analysis up to 24 months

**DOI:** 10.1590/1677-5449.010416

**Published:** 2019-01-30

**Authors:** Martin Andreas Geiger, Ana Terezinha Guillaumon

**Affiliations:** 1 Universidade Estadual de Campinas – UNICAMP, Hospital de Clínicas, Disciplina de Moléstias Vasculares, Campinas, SP, Brasil.

**Keywords:** stents, vascular patency, thrombosis

## Abstract

**Background:**

Primary stenting is a well-established treatment option for femoropopliteal arterial obstructive disease. There is a shortage of Brazilian studies of the subject.

**Objectives:**

To evaluate short and mid-term clinical and radiological outcomes in patients classified as Rutherford 3-6 and treated with stenting of femoropopliteal lesions.

**Methods:**

Analysis based on a prospectively populated database of patients treated from July 2012 to July 2015. The primary endpoint was primary patency. Secondary endpoints were clinical and ankle/brachial index changes. Target Vessel Revascularization, limb salvage rate and death, within a 24-month follow-up period.

**Results:**

64 patients were enrolled, including 61 TASC II A / B lesions (95%). The primary patency rates at 6, 12, and 24 months were 95.2%, 79.1% and 57.9%, respectively. Cox regression analysis revealed lower patency rates in patients with occlusive disease (hazard ratio [HR], 6.64; 95% confidence interval [CI], 1.52-28,99, p = 0.02), as well as patency loss about 6 times higher in TASC B than in TASC A patients ([HR], 5.95, 95% CI, 1.67-21.3, p = 0.0061). At 12 months, 90.38% of the patients remained asymptomatic. The limb salvage rate at 24 months was 94.3% (95% CI, 87.9-100%). Freedom from TVR at 24 months was 90.5% (95% CI 82.8-98.9%).

**Conclusions:**

Results of primary patency were compatible with international studies, despite the more advanced stage of the vascular disease observed in our group. Occlusive disease and complex lesions were both associated with worse outcomes.

## INTRODUCTION

 Peripheral arterial disease (PAD) affects almost 12% of the general population, 20% of whom are over the age of 65. 1 It is responsible for reducing patients’ functional capacity, deteriorating quality of life, and increasing the risk of limb loss. It is also associated with acute myocardial infarction, stroke, and death. 2

 Current treatment options include both open surgery and endovascular techniques. Those who are in favor of the conventional surgical approach emphasize the high patency rates over the long term and the durability of clinical improvement. However, this option can be related to high rates of morbidity and mortality, and requires considerable resources. Additionally, durability is directly dependent on constant surveillance of the graft, frequent outpatients visits and imaging exams such as vascular Doppler ultrasound, and can require repeated reinterventions. 3

 On the other hand, those who are in favor of the endovascular approach point to the lower morbidity and mortality rates, lower costs, and shorter durations of operations and hospital stays, when compared with conventional surgery. Additionally, even though there is a possibility of failure with endovascular techniques, in contrast to bypass surgery, subsequent surgical interventions are not compromised and the collateral arterial branches are preserved. 4

 Stents have undergone significant technological evolution over recent years and their use, which was previously limited to coronary interventions, now has applications in the femoropopliteal territory. This is the result of publications of controlled and randomized studies demonstrating increased primary patency of arteries that were previously treated with balloon angioplasty. 5

 The focus of studies is to determine which treatments offer the best cost-benefit ratio in terms of the materials used, such as stents, drug-eluting stents, or even other devices that could increase the patency rates of endovascular treatment. It should also be pointed out that, despite the intense interest in developing endovascular techniques, we still lack more robust epidemiological data, including risk factors and the characteristics of the disease and the patients, which could help not only in selection of interventions, but also in optimization of treatment as a whole. 

## OBJECTIVES

 The primary objective was to evaluate primary patency of the treated artery among patients with femoral artery PAD, treated with endovascular stent placement, from July 2012 to July 2015. The secondary objective was to evaluate improvements in the Rutherford classification and the ankle/brachial index (ABI), target-vessel revascularization (TVR), limb salvage rate, and mortality during the 24 months after the procedure. Target-vessel revascularization was defined as any percutaneous or open surgical intervention in the superficial femoral artery conducted because of lost patency or recurrence of ischemic symptoms in the limb, making it an index that illustrates the percentage of patients who required TVR after the initial intervention. 

## METHOD

 This is a single-center and single-armed longitudinal retrospective analysis based on a database that is populated prospectively (a longitudinal study with protocol) covering patients with PAD at the Hospital de Clínicas da Universidade Estadual de Campinas (UNICAMP), located in Campinas, SP, Brazil. The sample comprised symptomatic patients (Rutherford 3-6) treated with stenting in the femoropopliteal territory from July 2012 to July 2015. The popliteal territory was defined as up to P1, which corresponds to the proximal segment, running from the channel of the adductor muscles to the upper margin of the patella. The patients analyzed were treated with anterograde and retrograde access, using S.M.A.R.T. Control™ (Cordis, Miami Lakes, FL, United States) and Astron Pulsar (Biotronik AG, Buelach, Switzerland) stents. 

 Primary patency was defined as absence of significant intrastent stenosis or occlusion on Doppler ultrasound or arteriography with no need for any type of intervention. The criterion employed to define intrastent stenosis was a peak systolic velocity ratio greater than 2. 

### Inclusion criteria

 Patients were enrolled who had superficial femoral artery lesions treated by stenting, who had full clinical and imaging exam assessments in outpatients follow-up, had at least one of the three distal arteries patent on angiography, and were free from severe infections of the extremities. 

### Exclusion criteria

 Patients were excluded if they had proximal arterial disease, no runoff arteries, other etiologies such as trauma, arterites, acute arterial occlusion, severe infections of the extremities; prior open or endovascular surgery(ies) on the limb, conditions affecting the vascular access site, aneurysms or other diseases involving the popliteal artery, below-the-knee amputations, renal failure (creatinine clearance < 30 mL/min), limited life expectancy, coagulopathies, restrictions limiting use of antiplatelet drugs; incomplete medical records, missing description of the lesion, or missing imaging exams. 

 Descriptive statistics were calculated on the basis of the total number of patients assessable for each variable. For continuous variables, summary measures were calculated (mean, standard deviation, range). Categorical variables are presented in frequency tables (absolute and relative frequencies). For the purposes of comparative analysis, contingency tables were constructed containing absolute frequencies and percentages, and Pearson’s chi-square test was used to test the hypothesis of independence of variables. 

 Two-year survival was analyzed in relation to the variables loss of primary patency, limb salvage, and absence of TVR using the Kaplan-Meier estimator. Kaplan-Meier estimates were compared between groups using the log-rank test for the hypothesis of equal curves. 

 The data on time to lost primary patency were analyzed with respect to variables of interest using an adjusted Cox regression model. Covariates were selected using partial likelihood ratio tests. The assumption of proportional risks was tested using the graphical method with Schoenfeld standardized residuals and the respective tests of proportionality of risks associated with the adjusted model. All statistical analyses were conducted using R® version 3.3.1. 

## RESULTS

### Population data

 From July 2012 to July 2015, 92 patients were treated with endovascular stenting. Sixty-four of these patients were included in the study sample because complete medical records and all necessary data were available. The mean age of these patients was 68 years and 64% were female. Table 1 lists the comorbidities of the patients studied. One patient (2%) had limiting claudication (Rutherford 3), 11 (17%) had pain at rest (Rutherford 4), and 52 (81%) had trophic lesions (Rutherford 5 and 6). 

**Table 1:** Distribution by comorbidities.

**Comorbidities**	**n**	**%**
Smoking	41	64.06
Systemic arterial hypertension	58	90.62
Diabetes	48	75.00
Heart disease	4	6.25
Lung disease	1	1.56
Kidney disease	3	4.69
Dyslipidemia	36	56.25

total n = 64 patients.

Table 2 lists angiographic findings for each group before stenting. The results after 24 months were not available for 33 patients. The mean number of stents used per patient was one. Mean length of the stents used was 80 mm (range: 40-150 mm) and mean diameter was 5 mm (range: 4-6 mm). 

**Table 2:** Preoperative angiographic findings according to the Rutherford classification.

**Angiographic findings** ^*^		**Rutherford**				**p**
		**3**	**4**	**5**	**6**	
Total		1 (1.56%)	11 (17.19%) (17.19%)	51 (79.69%) (79.69%)	1 (1.56%)	--
TASC II	A	1 (1.56%)	6 (9.38%)	25 (39.06%)	0 (0)	0.81
	B	0 (0)	4 (6.25%)	24 (37.5%) (37.5%)	1 (1.56%) (1.56%)	
	C	0 (0)	1 (1.56%)	2 (3.13%)	0 (0)	
Lesion	Stenosis	1 (1.56%)	5 (7.81%)	18 (28.13%)	0 (0)	0.45
	Occlusion	0 (0)	6 (9.38%)	33 (51.56%)	1 (1.56%)	
Runoff arteries	1	0 (0)	7 (10.94%) (10.94%)	42 (65.63%)	1 (1.56%)	< 0.01
	2	0 (0)	4 (6.25%)	9 (14.06%)	0 (0)	
	3	1 (1.56%)	0 (0)	0 (0)	0 (0)	

* Categorical data are expressed as absolute values (%); total n = 64 patients.

**Figure 1:**
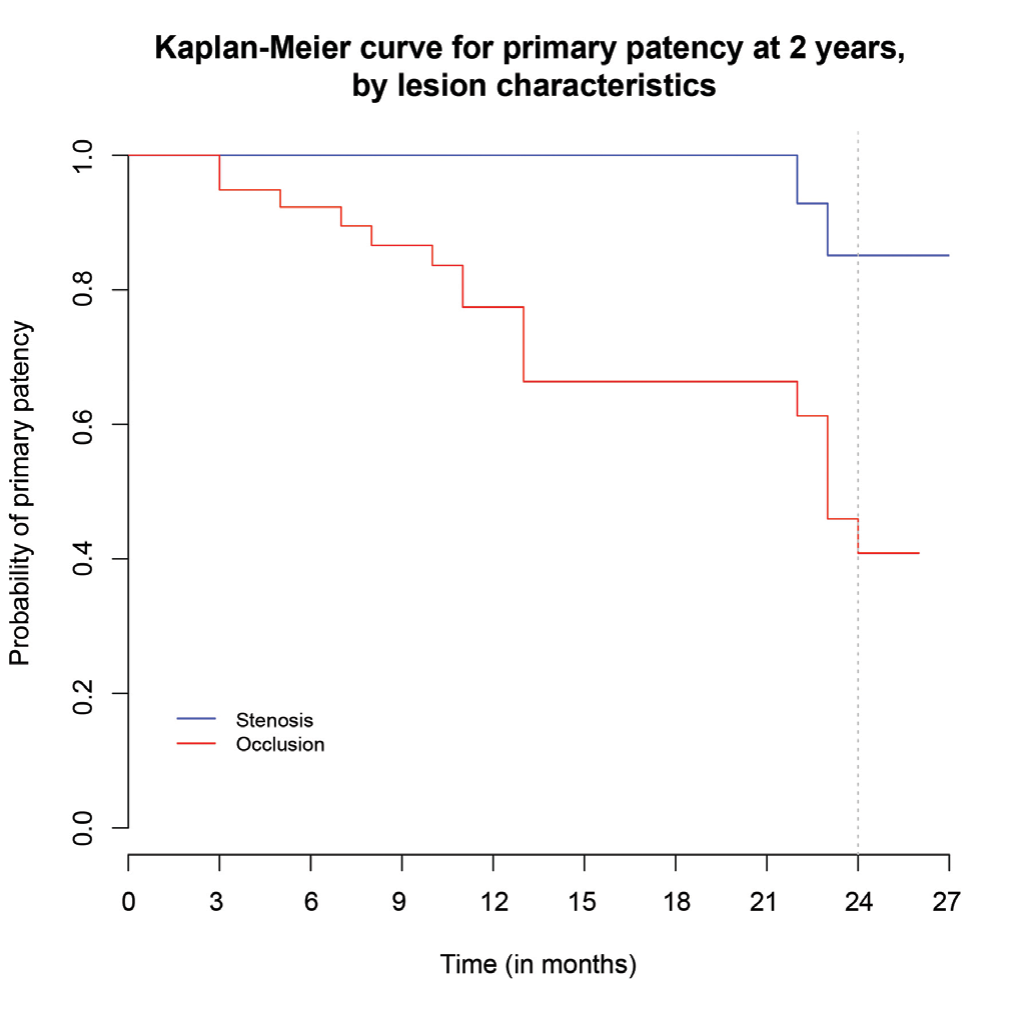
Kaplan-Meier curve illustrating primary patency by lesion characteristics (p = 0.00294).

**Figure 2:**
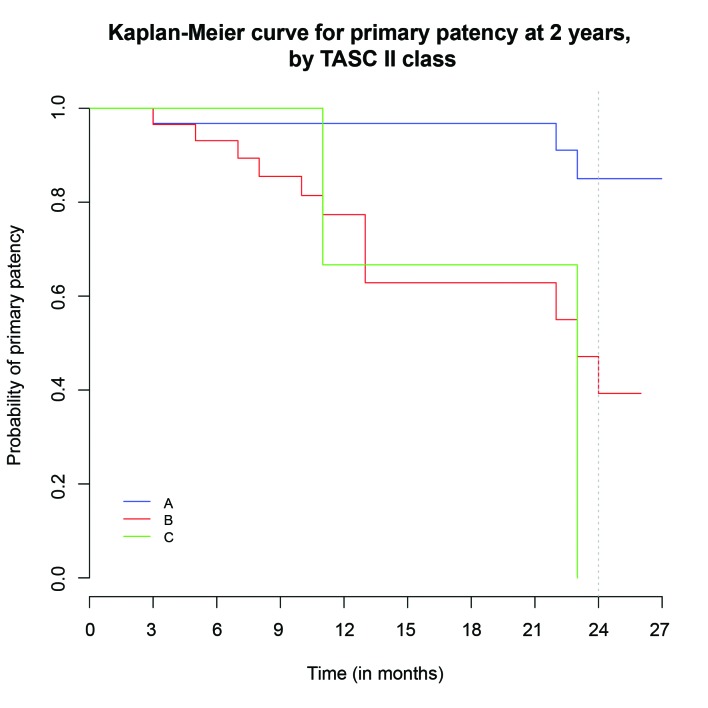
Kaplan-Meier curve illustrating primary patency in subsets (p = 0.0017).

 Sixty-two point five percent (62.5%) of the patients assessed had occlusive disease and two or more runoff arteries were present in just 21.87%. Presence of occlusive disease was associated with deterioration of patency (hazard ratio (HR), 6.64; 95% confidence interval [CI], 1.52-28.99; p = 0.02) ( Figure 1 ). Seventeen percent (17%) of these patients with occlusion were TASC A, 42% were TASC B and 3% were TASC C. The risk of patency loss for TASC B patients was around six times greater than for TASC A patients (HR, 5.95; 95%CI, 1.67-21.3; p = 0.0061) and around nine times greater for TASC C patients than for TASC A patients (HR, 9.35; 95%CI, 1.86-46.9; p = 0.0066) ( Figure 2 ). 

### Primary patency

**Figure 3:**
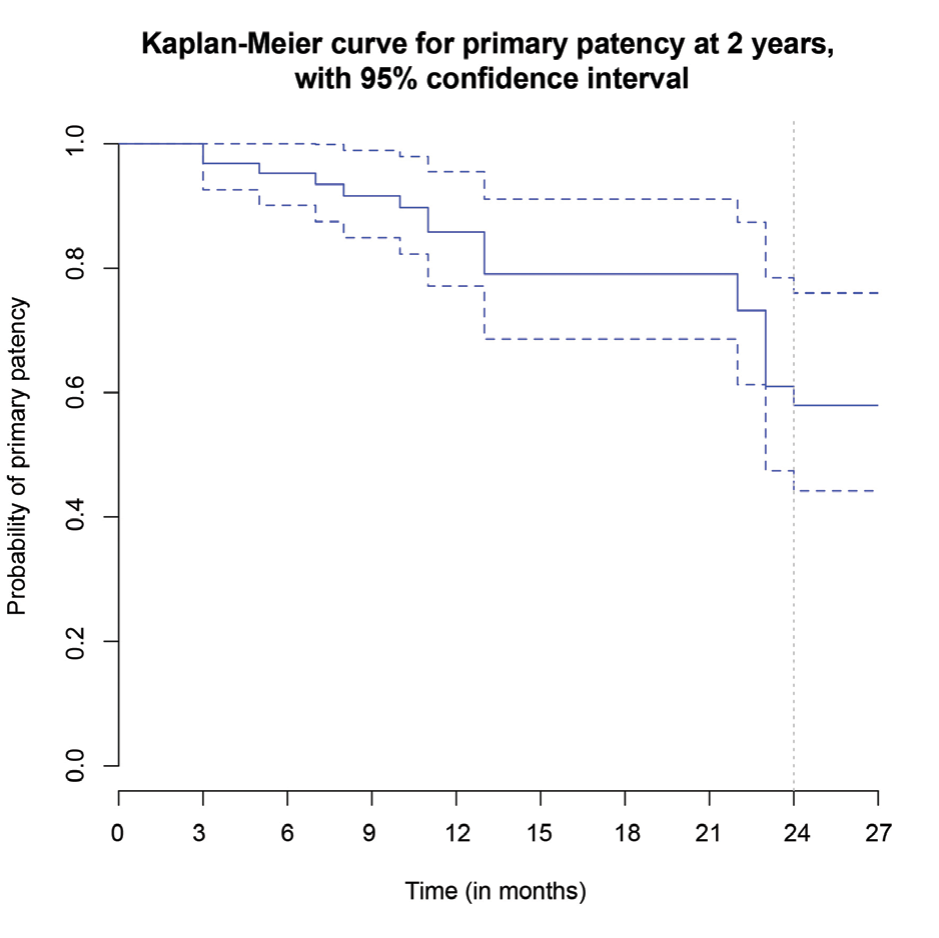
Kaplan-Meier curve illustrating primary patency. Broken lines indicate the 95% confidence interval.

**Figure 4:**
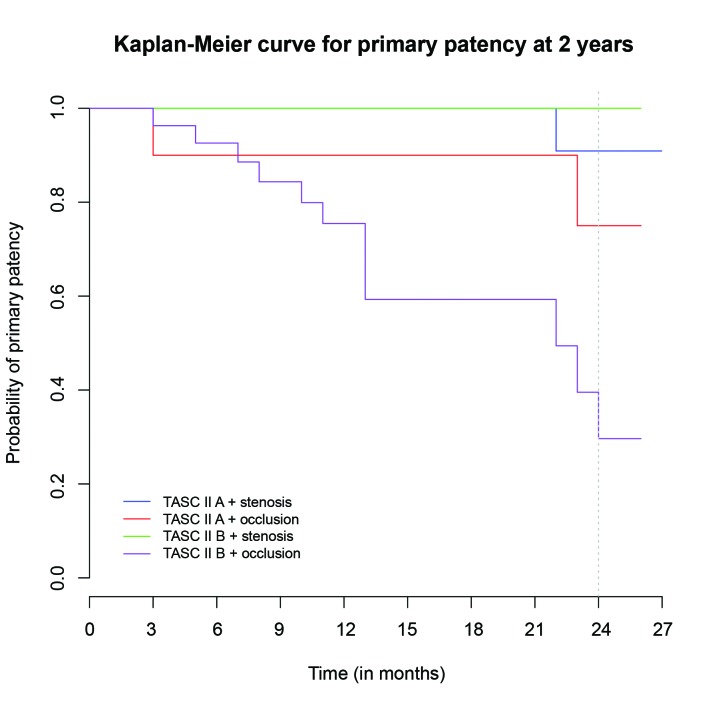
Kaplan-Meier curve illustrating primary patency in subsets (p = 0.00269).

 Primary patency was 95.2% (95%CI, 90.1-100%) at 6 months, 79.1% (95%CI, 68.6-91.1%) at 12 months and 57.9% (95%CI, 44.2-76%) at 24 months. In terms of the time to loss of patency, a fall in patency was observed up to 12 months after the procedure ( Figure 3 ). Analysis of patency by subsets revealed lower patency in TASC B patients with occlusive disease ( Figure 4 ). Patency in this subset was 59.3% (95%CI, 41.5-84.7%) at 12 months and 29.6% (95%CI, 12.4-71.2%) at 24 months. 

### Rutherford classification and ABI

 An improvement of at least one Rutherford PAD category was observed at 12 and 24 months. At 12 months, 90.38% of the patients analyzed remained asymptomatic and at 24 months 87.1% had remained stable in the same Rutherford classification category. 

 Mean ABI ranged from 0.47 (standard deviation [SD]: 0.15) preoperatively to 0.84 (SD, 0.23) during the postoperative period. There was a progressive reduction at 12 and 24 months, with values of 0.75 (SD, 0.22) and 0.67 (SD, 0.15), respectively. 

### Amputation and TVR rates

 The limb salvage rate at 24 months was 94.3% (95%CI, 87.9-100%). By 24 months, three major amputations had occurred as a result of disease progression. Two patients died for reasons unconnected to PAD. Five patients underwent additional revascularizations, some via open and some via endovascular methods. The time of reintervention ranged from 3 to 12 months after the original procedure. The rate of absence of TVR at 24 months was 90.5% (95%CI, 82.8-98.9%). 

 One of the patients who underwent an amputation had suffered intense refractory pain during the first month after stenting associated with infection. The second patient presented at the emergency room 1 month after stenting with an extensive lower limb infection up to the thigh. 

## DISCUSSION

 Stenting in the femoropopliteal territory is common in situations of elastic remodeling of the artery or dissections with hemodynamic repercussions after angioplasty. 6 Several studies have demonstrated the superiority of stenting over angioplasty without stent placement, with better clinical and radiological results at 6 and 12 months. 7 There are few Brazilian studies. Our objective was to conduct a review of patients treated at our institution and followed-up for 24 months, identifying not only the characteristics and results of our treatment, but also the characteristics of patients with PAD who are treated at a public Brazilian institution. 

 The majority of large studies published to date include patients with symptomatic PAOD, whose predominant symptom is intermittent claudication. 8 ^-^ 11 In such studies there are also significant numbers of patients with more than one runoff artery, which directly impacts the patency and TVR results. In contrast, our sample contained a large majority of patients with advanced critical ischemia (Rutherford 5 and 6). 

 The majority of patients seen at the Vascular Diseases Clinic are referred to us by the Brazilian National Health Service (SUS - Sistema Único de Saúde). They have not been investigated in detail and a more consistent pre-assessment is needed. The majority of these patients are still smoking, hypertensive, poorly-controlled diabetics, with unstable control of cholesterol and triglycerides. Since many of these patients’ medical diagnoses are made late, the first symptoms manifest are trophic lesions. 10 ^,^ 12 These patients already have critical ischemia, advanced disease, and compromised runoff arteries, and are poorly stratified from cardiological, renal, and pulmonary perspectives. This is evident in the demographic results of our study. We understand that no prior diagnosis or treatment of pulmonary and renal comorbidities occurs, since many of these patients are only treated after admission to hospital. 

 We chose a 24-month analysis period using a protocol for recording care provided to patients treated at our service. There is no doubt that standardization of nitinol stents facilitated the comparisons and analyses conducted. 13 ^,^ 14

 Nitinol stents were introduced to improve durability and patency in treated superficial femoral arteries. 5 Nitinol has unique properties, such as its capacity to return to its original shape after deformation, known as elastic memory. This characteristic, combined with its biocompatibility, has made nitinol the material of choice for stents. 15 It should be pointed out that all systematic reviews of the subject analyze earlier studies in conjunction, from when stainless steel stents were still being used and discussed. Non-uniform analyses are employed. However, it is known that stents made from nitinol, which is now the material of choice, exhibit behavior that is distinct both in terms of the intrinsic characteristics of the lesion and of the extrinsic characteristics of the femoral artery. 15 ^-^ 17

 Among the patients evaluated in this study, a downward trend in primary patency was observed over the first 12 first months. This tendency has also been reported in other studies. 18 ^-^ 21

 Myointimal hyperplasia and intrastent stenosis are still the Achilles heel of angioplasty with stenting. Over recent years, studies have investigated drug eluting stents, drug eluting balloons, and covered stents. The objective of these alternative technologies lies in the theoretical advantage of preventing these events, increasing primary patency. 22 However, it is notable that such studies are still limited to evaluating the use of these technologies for non-complex lesions with little calcification. 23 ^,^ 24

 The values for primary patency observed among our patients were similar to reports in the literature. 12 ^,^ 15 ^,^ 17 ^,^ 18 ^,^ 20 We attribute this result, i.e., the better 12-month patency, to the types of lesion treated. In our sample, patients were predominantly classed as TASC A and TASC B; just three of the patients treated were TASC C, according to the classification used in the TASC II multicenter study. 12 ^,^ 25 Comparison by subsets revealed lower patency rates among patients with occlusive and longer lesions (TASC B). 

 Currently, there is a worldwide tendency to initially attempt endovascular management, irrespective of the characteristics of the lesion. 9 ^,^ 26 This trend, however, respects criteria that are also adopted at our service, such as avoiding release of the stent in areas proximal of the superficial femoral artery, since this risks occluding the deep femoral artery, and also of avoiding treatment with stents in lesions that extend beyond the knee joint line. Many specialists consider the knee a hostile region for endovascular treatment because of the considerable mobility of the popliteal artery, which imposes stress on the stent material, breaking it and compromising patency. Newer stents, with different radial strength and flexibility, have been developed for use in areas with extreme mobility demands, but they are still undergoing long-term assessment. 27 We consider that patients with involvement of the popliteal artery in the joint region are candidates for open surgery. 

 Endovascular treatment as the first choice is still justified by the fact that it offers a less invasive repair, lower risk of infection of the surgical wound, low rates of complications, preserving donor and recipient areas of the artery, and shorter operation duration. 7

 At 12 and 24 month follow-ups, 90.38% and 87.1% of patients, respectively, maintained their post-treatment clinical improvement. These results are backed up by the low rate of TVR at 24 months. This is attributed to the conduct employed at the service studied, which requires reintervention only when two criteria are considered: recurrence of symptoms and presence of restenosis in the area treated, identified by ultrasound. This policy is aimed at the primary objective of revascularization, which is to improve the patient’s symptoms and save the limb. In our analysis, just three patients had limbs amputated, because of advanced infectious processes involving trophic ulcers in diabetic patients. 

 The ABI is an important predictor for patency follow-up. 12 ^,^ 25 ^,^ 28 However, it is known that many patients have characteristics that restrict measurement of this parameter. Patients with diabetes mellitus may have calcification of the distal arteries of the lower limbs, resulting in elevated ABI values. We chose to assess ABI in terms of follow-up, and not the absolute value. The satisfactory results of this study are subject to limitations, since just 48% of the original patient sample was followed for 24 months. 

## CONCLUSIONS

 Primary stenting for treatment of the superficial femoral artery is still a treatment option with good clinical and radiological results over the short and medium term. Our results were in line with those reported in large-scale studies and were superior to results reported for treatment with angioplasty alone, without stenting. Longer, more complex, and occlusive lesions exhibit worse results when treated. However, the primary objective of treatment, which is limb salvage, is achieved in the great majority of cases. 
