# Supplementary material for: Primary stenting for femoropopliteal peripheral arterial disease: analysis up to 24 months
Source: J Vasc Bras. 2019 Jan 30;18:e20160104. doi: 10.1590/1677-5449.010416 (PMC6542323; doi:10.1590/1677-5449.010416)
Supplement: Translation

# Translation: Tratamento da doença arterial obstrutiva periférica em território femoropoplíteo com stent primário: análise em até 24 meses

**Authors:** Martin Andreas Geiger, Ana Terezinha Guillaumon

**Affiliations:** 1 Universidade Estadual de Campinas – UNICAMP, Hospital de Clínicas, Disciplina de Moléstias Vasculares, Campinas, SP, Brasil.

**Keywords:** stents, patência vascular, trombose

## Abstract

**Contexto:**

O stent primário é uma opção de tratamento bem estabelecida para a doença arterial periférica em território femoropoplíteo. Estudos nacionais são escassos.

**Objetivos:**

Avaliar desfechos clínicos e radiológicos em curto e médio prazo em pacientes classificados como Rutherford 3-6, tratados com o uso de stent em lesões femoropoplíteas.

**Métodos:**

A análise foi realizada com base em um banco de dados prospectivamente mantido de doentes tratados entre julho de 2012 e julho de 2015. O objetivo primário foi a perviedade. Os objetivos secundários foram melhora na classificação de Rutherford, índice tornozelo/braço, revascularização do vaso-alvo, taxa de salvamento do membro e óbito em até 24 meses.

**Resultados:**

Foram incluídos 64 pacientes, sendo 61 com lesões TASC II A/B (95%). A taxa de perviedade primária em 6, 12 e 24 meses foi de 95,2%, 79,1% e 57,9%, respectivamente. A análise de regressão de Cox revelou uma menor perviedade em pacientes com doença oclusiva (RR, 6,64, IC 95%, 1,52-28,99, p = 0,02), bem como uma perda de perviedade cerca de seis vezes maior em doentes TASC B do que TASC A (RR, 5,95, IC 95%, 1,67-21,3, p = 0,0061). Em 12 meses, 90,38% dos doentes permaneceram assintomáticos. A taxa de salvamento do membro em 24 meses foi de 94,3% (IC 95%, 87,9-100%). A ausência de revascularização do vaso-alvo em 24 meses foi de 90,5% (IC 95%, 82,8-98,9%).

**Conclusões:**

Os resultados foram compatíveis com estudos internacionais, apesar do estágio mais avançado da doença vascular observada em nosso grupo. Piores desfechos foram associados a doença oclusiva e lesões complexas.

## INTRODUÇÃO

 A doença arterial periférica (DAP) afeta quase 12% da população geral, sendo que 20% dos indivíduos têm idade superior a 65 anos 1 . Ela é responsável pela diminuição da capacidade funcional do doente, deterioração da qualidade de vida e aumento do risco de perda de membro. Está associada a infarto agudo do miocárdio, acidente vascular cerebral e morte 2 . 

 Atualmente, tanto as cirurgias abertas quanto as técnicas endovasculares são consideradas opções terapêuticas. Aqueles que defendem a abordagem cirúrgica convencional enfatizam as altas taxas de perviedade em longo prazo e a durabilidade da melhora clínica. No entanto, essa opção terapêutica pode estar relacionada a uma alta morbimortalidade, bem como demandar recursos substanciais. Além disso, tal durabilidade depende diretamente de uma constante vigilância do enxerto, de retornos ambulatoriais frequentes e de exames de imagem como o ultrassom Doppler vascular, além de requerer, muitas vezes, reintervenções repetidas 3 . 

 Os defensores da abordagem endovascular, por outro lado, apontam para as baixas taxas de morbimortalidade, menor custo e redução do tempo operatório e de internação, quando comparada à cirurgia convencional. Além disso, mesmo com uma possível falha na abordagem endovascular, uma posterior cirurgia não fica comprometida, ao contrário da cirurgia com pontes, preservando ramos arteriais colaterais 4 . 

 Os stents representaram uma significativa evolução tecnológica nos últimos anos e sua utilização, anteriormente limitada a intervenções coronarianas, ganhou espaço no território femoropoplíteo. Isso se deve a publicações de estudos controlados e randomizados demonstrando um aumento na perviedade primária das artérias anteriormente angioplastadas apenas com balão 5 . 

 O foco dos estudos é estabelecer o tratamento com o melhor custo-benefício em relação aos materiais utilizados, como stents, stents farmacológicos, balões farmacológicos ou mesmo outros dispositivos que venham a aumentar a taxa de perviedade do tratamento endovascular. Ressalta-se também que, apesar da grande preocupação no desenvolvimento da técnica endovascular, carecemos ainda de dados epidemiológicos robustos, incluindo fatores de risco, características da doença e do doente, que poderiam vir a ajudar não só na escolha, mas também na otimização do tratamento como um todo. 

## OBJETIVOS

 O objetivo primário foi avaliar a perviedade primária da artéria tratada nos doentes com DAP de artéria femoral, submetidos a abordagem endovascular com o uso de stent, de julho de 2012 a julho de 2015. O objetivo secundário foi avaliar melhora na classificação de Rutherford e no índice tornozelo/braço (ITB), revascularização do vaso-alvo (RVA), taxa de salvamento do membro e óbito durante o acompanhamento em até 24 meses do procedimento. RVA foi considerada qualquer intervenção percutânea ou cirurgia aberta da artéria femoral superficial realizada em razão de perda da perviedade ou recorrência dos sintomas isquêmicos no membro, traduzindo-se como o índice que demonstra a porcentagem de doentes que necessitaram de uma nova RVA. 

## MÉTODO

 Trata-se de uma análise longitudinal retrospectiva em centro único, braço único, tendo como base o banco de dados alimentado prospectivamente (estudo longitudinal sob protocolo) de doentes com DAP do Hospital de Clínicas da Universidade Estadual de Campinas (UNICAMP), localizado em Campinas, SP, Brasil. Foram avaliados doentes sintomáticos (Rutherford 3-6) tratados com o uso de stent em território femoropoplíteo entre julho de 2012 e julho de 2015. O território poplíteo foi considerado até P1, que corresponde ao segmento proximal, indo do canal dos músculos adutores até a borda superior da patela. Foram avaliados doentes submetidos a acesso anterógrado e retrógrado com uso de stent S.M.A.R.T. Control™ (Cordis, Miami Lakes, FL, EUA) e Astron Pulsar (Biotronik AG, Buelach, Suíça). 

 A perviedade primária foi definida como ausência de estenose significativa intra-stent ou oclusão ao ultrassom Doppler ou arteriografia na ausência de qualquer intervenção. Utilizou-se como critério para a definição de estenose intra-stent uma relação de velocidade de pico sistólico maior do que 2. 

### Critérios de inclusão

 Foram incluídos doentes com lesão em artéria femoral superficial tratados com stent, que apresentavam avaliação clínica completa e de imagem no acompanhamento ambulatorial de seguimento, com pelo menos uma das três artérias distais pérvias ao exame angiográfico, sem infecção grave nas extremidades. 

### Critérios de exclusão

 Foram excluídos aqueles com doença arterial proximal, ausência de artérias de deflúvio, outras etiologias como trauma, arterites, oclusão arterial aguda e infecções graves de extremidades; cirurgias prévias abertas ou endovasculares de extremidade, alterações no local de acesso vascular, aneurismas ou outras doenças na artéria poplítea, amputações infrapatelares, insuficiência renal (*clearence* de creatinina < 30 mL/min), expectativa de vida limitada, coagulopatias, restrições ao uso de antiplaquetários; prontuário incompleto, ausência de caracterização da lesão, assim como ausência de exames de imagem. 

 As medidas descritivas foram calculadas respeitando o total de doentes avaliáveis em cada variável. Para as variáveis contínuas, foram obtidas as medidas resumo (média, desvio padrão, máximo e mínimo). As variáveis categóricas foram apresentadas em tabelas de frequência (frequência absoluta e relativa). Para análise cruzada, foram feitas tabelas de contingência com frequência absoluta e porcentagem, e o teste qui-quadrado de Pearson foi realizado para a hipótese de independência entre as variáveis. 

 Para avaliar a sobrevida no período de 2 anos com relação às variáveis perda de perviedade primária, salvamento de membro e ausência de RVA, foi utilizado o estimador de Kaplan-Meier. Para a comparação das estimativas obtidas via Kaplan-Meier entre grupos, foi feito o teste de log-rank para a hipótese de igualdade das curvas. 

 Para a análise dos dados provenientes do tempo até a perda da perviedade primária com relação a variáveis de interesse, foi ajustado o modelo de regressão de Cox. A seleção das covariáveis foi feita a partir do teste da razão de verossimilhanças parcial. Para checar a suposição de existência de riscos proporcionais, foi utilizado o método gráfico com resíduos padronizados de Schoenfeld, bem como os respectivos testes da proporcionalidade dos riscos associados ao modelo ajustado. Toda a análise estatística foi feita no software R®, versão 3.3.1. 

## RESULTADOS

### Dados populacionais

 Entre julho de 2012 e julho de 2015, 92 doentes foram submetidos a abordagem endovascular com implante de stent. Desses, 64 doentes foram incluídos por apresentarem prontuários e dados completos. A idade média dos doentes foi de 68 anos, e 64% eram do sexo feminino. A Tabela 1 apresenta as comorbidades dos doentes estudados. Claudicação limitante (Rutherford 3) estava presente em um doente (2%), dor de repouso (Rutherford 4) em 11 doentes (17%), e lesão trófica (Rutherford 5 e 6) em 52 doentes (81%). 

**Tabela 1:** Distribuição de acordo com comorbidades.

**Comorbidades**	**n**	**%**
Tabagismo	41	64,06
Hipertensão arterial sistêmica	58	90,62
Diabetes	48	75,00
Doença cardíaca	4	6,25
Doença pulmonar	1	1,56
Doença renal	3	4,69
Dislipidemia	36	56,25

n total = 64 doentes.

 A Tabela 2 apresenta os achados angiográficos em cada grupo antes do implante do stent. Os resultados em 24 meses não estavam disponíveis em 33 doentes. Foi utilizada uma média de um stent implantado por doente. O comprimento médio dos stents implantados foi de 80 mm (variação, 40-150) e o diâmetro médio foi de 5 mm (variação, 4-6). 

**Tabela 2:** Achados angiográficos pré-operatórios de acordo com a classificação de Rutherford.

**Achados angiográficos** ^*^		**Rutherford**				**Valor de p**
		**3**	**4**	**5**	**6**	
Total		1 (1,56%)	11 (17,19%) (17,19%)	51 (79,69%) (79,69%)	1 (1,56%)	--
TASC II	A	1 (1,56%)	6 (9,38%)	25 (39,06%)	0 (0)	0,81
	B	0 (0)	4 (6,25%)	24 (37,5%) (37,5%)	1 (1,56%) (1,56%)	
	C	0 (0)	1 (1,56%)	2 (3,13%)	0 (0)	
Lesão	Estenose	1 (1,56%)	5 (7,81%)	18 (28,13%)	0 (0)	0,45
	Oclusão	0 (0)	6 (9,38%)	33 (51,56%)	1 (1,56%)	
*Runoff*	1	0 (0)	7 (10,94%) (10,94%)	42 (65,63%)	1 (1,56%)	< 0,01
	2	0 (0)	4 (6,25%)	9 (14,06%)	0 (0)	
	3	1 (1,56%)	0 (0)	0 (0)	0 (0)	

* Os dados categóricos são apresentados como número (%); n total = 64 doentes.

 Dos doentes avaliados, 62,5% apresentavam lesão oclusiva, sendo que, em apenas 21,87%, duas ou mais artérias de deflúvio estavam presentes. A presença de lesão oclusiva esteve associada a piora da perviedade [razão de risco (RR), 6,64; intervalo de confiança (IC) de 95%, 1,52-28,99; p = 0,02] ( Figura 1 ). Desses doentes com oclusão, 17% eram TASC A, 42% eram TASC B e 3% eram TASC C. O risco de perda de perviedade em doentes TASC B foi cerca de seis vezes maior se comparados a doentes TASC A (RR, 5,95; IC 95%, 1,67-21,3; p = 0,0061) e cerca de nove vezes maior em doentes TASC C se comparados a doentes TASC A (RR, 9,35; IC 95%, 1,86-46,9; p = 0,0066) ( Figura 2 ). 

**Figura 1:** Curva de Kaplan-Meier demonstrando a perviedade primária por característica da lesão (p = 0,00294).

**Figura 2:** Curva de Kaplan-Meier demonstrando a perviedade primária nos subgrupos (p = 0,0017).

### Perviedade primária

 A perviedade primária foi de 95,2% (IC 95%, 90,1-100%) em 6 meses, de 79,1% (IC 95%, 68,6-91,1%) em 12 meses e de 57,9% (IC 95%, 44,2-76%) em 24 meses. Quanto ao tempo da perda de perviedade, um declínio foi observado até 12 meses do procedimento ( Figura 3 ). Na avaliação da perviedade por subgrupos, observou-se uma menor perviedade em doentes TASC B com lesão oclusiva ( Figura 4 ). A perviedade nesse subgrupo foi de 59,3% (IC 95%, 41,5-84,7%) em 12 meses e de 29,6% (IC 95%, 12,4-71,2%) em 24 meses. 

**Figura 3:** Curva de Kaplan-Meier demonstrando a perviedade primária. As linhas tracejadas indicam o intervalo de confiança de 95%.

**Figura 4:** Curva de Kaplan-Meier demonstrando a perviedade primária nos subgrupos (p = 0,00269).

### Classificação de Rutherford e ITB

 Uma melhora de pelo menos uma categoria de DAP de Rutherford foi observada em 12 e 24 meses. Em 12 meses, 90,38% dos doentes avaliados mantiveram-se assintomáticos e, em 24 meses, 87,1% dos doentes mantiveram-se estáveis na mesma categoria na classificação de Rutherford. 

 O ITB variou, em média, de 0,47 (desvio padrão de 0,15) no pré-operatório para 0,84 [desvio padrão (DP), 0,23] no pós-operatório. Houve uma diminuição progressiva em 12 e 24 meses, com valores de 0,75 (DP, 0,22) e 0,67 (DP, 0,15), respectivamente. 

### Taxa de amputação e RVA

 A taxa de salvamento do membro em 24 meses foi de 94,3% (IC 95%, 87,9-100%). Em 24 meses, ocorreram três amputações maiores decorrentes da evolução da doença. Dois doentes evoluíram a óbito não decorrente da DAP. Cinco doentes foram submetidos a nova revascularização tanto aberta como endovascular. O período de reintervenção foi de 3 a 12 meses após o primeiro procedimento. A ausência de RVA em 24 meses foi de 90,5% (IC 95%, 82,8-98,9%). 

 Dos doentes que foram submetidos a amputação, um apresentou dor intensa refratária no primeiro mês de pós-operatório associada à infecção. O segundo doente buscou o pronto-socorro após 1 mês com infecção extensa em membro inferior até a coxa. 

## DISCUSSÃO

 O uso do stent no território femoropoplíteo é comum em situações com remodelamento elástico da artéria ou dissecções com repercussão hemodinâmica após a angioplastia 6 . Diversos estudos têm demonstrado uma superioridade do stent sobre a angioplastia sem implante de stent, com melhores resultados clínicos e radiológicos em 6 e 12 meses 7 . Estudos nacionais são escassos. Nosso objetivo foi realizar uma revisão dos doentes tratados em nossa instituição acompanhados por até 24 meses, identificando não só características e resultados do nosso tratamento, mas também características do doente com DAP que chega a uma instituição pública nacional para ser tratado. 

 A maioria dos grandes estudos publicados até o momento incluem em sua casuística doentes com DAOP sintomáticos, apresentando claudicação intermitente como sintoma predominante 8 ^-^ 11 . Nesses estudos, observa-se ainda um número significativo de doentes com mais de uma artéria de deflúvio, com impacto direto no resultado de perviedade e RVA. Em contrapartida, notou-se em nossa amostra uma ampla maioria de doentes com isquemia crítica avançada (Rutherford 5 e 6). 

 A maioria dos doentes que chegam ao Ambulatório de Moléstias Vasculares são oriundos do Sistema Único de Saúde. Carecem de investigação mais detalhada, assim como de uma avaliação prévia mais consistente. São doentes, na sua maioria, tabagistas ativos, hipertensos, diabéticos mal controlados e dislipidêmicos com controle precário de colesterol e triglicérides. Muitos desses doentes, por terem um diagnóstico médico tardio, manifestam a lesão trófica como primeiro sintoma 10 ^,^ 12 . São doentes já com isquemia crítica, doença avançada e artérias de deflúvio comprometidas, mal estratificados do ponto de vista cardiológico, renal e pulmonar. Essa constatação fica evidente nos resultados demográficos do nosso estudo. Acreditamos que não exista um diagnóstico e tratamento prévio das comorbidades pulmonares e renais, já que muitos desses doentes são tratados apenas na internação hospitalar. 

 Optou-se pela escolha do período de até 24 meses de análise com um protocolo de atendimento e registro de doentes tratados no serviço. É inegável que a padronização de stents de nitinol facilitou as comparações e análises realizadas 13 ^,^ 14 . 

 Os stents de nitinol surgiram como uma melhoria relacionada à durabilidade e à perviedade da artéria femoral superficial tratada 5 . O nitinol apresenta propriedades únicas, como a capacidade de voltar à sua forma original após a deformação, chamada de memória elástica. Essa característica, unida à biocompatibilidade, tem feito do stent de nitinol o material de escolha 15 . Vale ressaltar que todas as revisões sistemáticas sobre o tema analisam de maneira conjunta estudos mais antigos, quando o uso de stent de aço inoxidável ainda era preconizado e discutido. Análises de heterogeneidade são aplicadas. Entretanto, sabe-se que o stent de nitinol, atualmente o material de escolha, apresenta comportamento distinto tanto com relação às características intrínsecas da lesão como às características extrínsecas da artéria femoral 15 ^-^ 17 . 

 Nos doentes avaliados neste estudo, um declínio da perviedade primária foi observado nos 12 primeiros meses. Essa tendência também é apresentada em outros estudos reportados 18 ^-^ 21 . 

 A hiperplasia miointimal e a estenose intra-stent permanecem sendo o calcanhar de Aquiles da angioplastia com implante de stent. Nos últimos anos, estudos têm avaliado o uso do stent farmacológico, do balão farmacológico e do stent revestido. O objetivo dessas tecnologias alternativas encontra-se na teórica vantagem de inibirem os eventos mencionados, aumentando a perviedade primária 22 . Observa-se, entretanto, que esses estudos ainda se limitam a avaliar o uso das tecnologias em lesões não complexas e pouco calcificadas 23 ^,^ 24 . 

 A perviedade primária observada em nossos doentes apresentou valores similares aos observados na literatura 12 ^,^ 15 ^,^ 17 ^,^ 18 ^,^ 20 . Atribuímos esse resultado, isto é, a melhor perviedade em 12 meses, ao tipo de lesão tratada. Em nossa amostra, os doentes eram predominantemente TASC A e TASC B; apenas três doentes tratados eram TASC C, conforme a classificação do estudo multicêntrico TASC II 12 ^,^ 25 . Na comparação por subgrupos, observou-se uma menor perviedade em doentes com lesões oclusivas e mais longas (TASC B). 

 Atualmente, observa-se uma tendência mundial à abordagem endovascular inicial, independentemente da característica da lesão 9 ^,^ 26 . Essa verdade, entretanto, respeita características também adotadas em nosso serviço como evitar a liberação de stent em região proximal da artéria femoral superficial, com o risco de se ocluir a artéria femoral profunda, assim como evitar o tratamento com stent em lesões que se estendam além da linha articular do joelho. A região articular é considerada por muitos especialistas como uma área hostil ao tratamento endovascular pela grande mobilidade da artéria poplítea, que ocasiona estresse do material do stent e quebra, comprometendo a perviedade. Novos stents, com diferente força radial e flexibilidade, têm sido desenvolvidos para uso em região de extrema mobilidade, mas ainda estão sob avaliação de longo prazo 27 . Consideramos os doentes com acometimento de artéria poplítea em região articular candidatos à cirurgia aberta. 

 A abordagem endovascular como primeira escolha pauta-se ainda na justificativa de apresentar correção menos invasiva, menor risco de infecção na ferida operatória, baixas taxas de complicação preservando as áreas doadoras e receptoras da artéria, assim como tempo operatório menor 7 . 

 Durante o acompanhamento de 12 e 24 meses, os doentes mantiveram a melhora clínica pós-tratamento de 90,38% e 87,1%, respectivamente. Esses resultados são reforçados pela baixa taxa de RVA nos 24 meses avaliados. Atribui-se isso à conduta preconizada no serviço, que estabelece reintervenção no doente apenas quando dois critérios são considerados: recorrência dos sintomas e presença de reestenose no local tratado ao ultrassom. Essa conduta contempla o objetivo primário da revascularização, que é melhorar a sintomatologia do doente e preservar o membro. Em nossa análise, apenas três doentes tiveram seus membros amputados, em decorrência de processos infecciosos avançados nas lesões tróficas em pacientes diabéticos. 

 O ITB é um importante preditor do acompanhamento da perviedade 12 ^,^ 25 ^,^ 28 . Sabe-se, entretanto, que muitos doentes apresentam limitação para mensuração desse parâmetro. Enfermos com diabetes melito podem apresentar calcificação das artérias distais dos membros inferiores, resultando em valores elevados. Optamos por avaliar o ITB em termos de acompanhamento, e não de valor absoluto. Os resultados satisfatórios deste estudo acima mencionados apresentam limitações, pois apenas 48% dos doentes originais foram monitorados por 24 meses. 

## CONCLUSÃO

 O tratamento da artéria femoral superficial com stent primário permanece sendo uma opção de tratamento com resultados clínicos e radiológicos bons em curto e médio prazo. Nossos resultados apresentaram-se conforme os descritos em grandes estudos, sendo superiores aos resultados relatados no tratamento somente com angioplastia sem implante de stent. Lesões mais longas, complexas e oclusivas apresentam piores resultados quando tratadas. Entretanto, o objetivo primário do tratamento, que é o salvamento do membro, é alcançado na grande maioria dos casos.
